# 1,5-Dimethyl-2-phenyl-4-[phenyl(pyri­din-2-ylamino)methyl]-1*H*-pyrazol-3(2*H*)-one

**DOI:** 10.1107/S1600536812042936

**Published:** 2012-10-20

**Authors:** K. Krishnakumar, S. Franklin, G. Venkatesa Prabhu, T. Balasubramanian

**Affiliations:** a Department of Chemistry, National Institute of Technology, Tiruchirappalli 620 015, Tamilnadu, India; b Department of Physics, Bishop Heber College (Autonomous), Tiruchirappalli 620 017, Tamilnadu, India; c Department of Physics, National Institute of Technology, Tiruchirappalli 620 015, Tamilnadu, India

## Abstract

In the title compound, C_23_H_22_N_4_O, the pyrazole ring makes dihedral angles of 45.57 (11)° with the attached phenyl ring, and 83.98 (10) and 67.85 (10) °, respectively, with the other phenyl ring and the pyridyl ring. The pyridyl ring makes a dihedral angle of 80.15 (10)° with the adjacent phenyl ring. In the crystal, N—H⋯O hydrogen bonds supplemented by weak C—H⋯O hydrogen bonds link the mol­ecules into chains which run parallel to the *a*-axis direction.

## Related literature

For the origin of the material studied, see: Vijayan (1971 ▶); Singh & Vijayan (1973 ▶). For related structures, see: Singh & Vijayan (1974 ▶, 1976 ▶); Tordjman *et al.* (1991 ▶); Yadav *et al.* (2003 ▶); Li & Zhang (2004 ▶); Wen (2005 ▶); Sun *et al.* (2007 ▶).
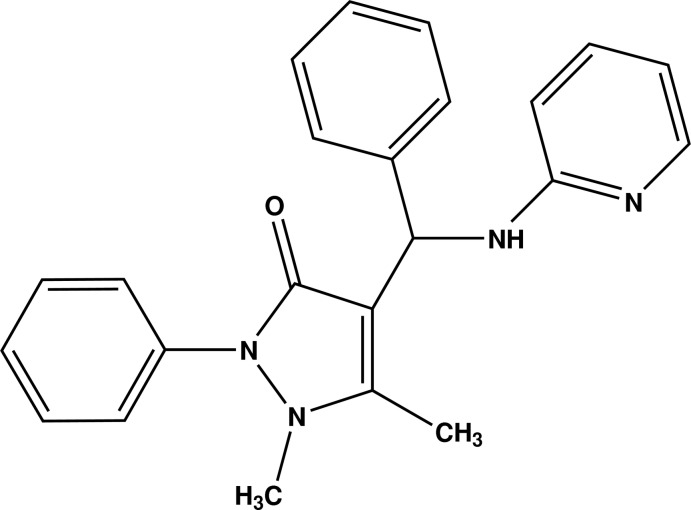


## Experimental

### 

#### Crystal data

C_23_H_22_N_4_O
*M* *_r_* = 370.45
Orthorhombic, 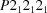

*a* = 5.701 (5) Å
*b* = 12.485 (5) Å
*c* = 26.736 (5) Å
*V* = 1903.0 (19) Å^3^
*Z* = 4
Mo *K*α radiation
μ = 0.08 mm^−1^
*T* = 273 K
0.3 × 0.2 × 0.2 mm

#### Data collection

Bruker Kappa APEXII CCD diffractometer
Absorption correction: multi-scan (*SADABS*; Bruker, 1999 ▶) *T*_min_ = 0.976, *T*_max_ = 0.984
14432 measured reflections
2959 independent reflections
2337 reflections with *I* > 2σ(*I*)
*R*_int_ = 0.036

#### Refinement

*R*[*F* ^2^ > 2σ(*F* ^2^)] = 0.040
*wR*(*F* ^2^) = 0.095
*S* = 1.01
2959 reflections
259 parameters
H atoms treated by a mixture of independent and constrained refinement
Δρ_max_ = 0.18 e Å^−3^
Δρ_min_ = −0.19 e Å^−3^

### 

Data collection: *APEX2* (Bruker, 2004 ▶); cell refinement: *APEX2* and *SAINT-Plus* (Bruker, 2004 ▶); data reduction: *SAINT-Plus* and *XPREP* (Bruker, 2004 ▶); program(s) used to solve structure: *SHELXS97* (Sheldrick, 2008 ▶); program(s) used to refine structure: *SHELXL97* (Sheldrick, 2008 ▶); molecular graphics: *ORTEP-3 for Windows* (Farrugia, 1997 ▶) and *Mercury* (Macrae *et al.*, 2006 ▶); software used to prepare material for publication: *PLATON* (Spek, 2009) ▶.

## Supplementary Material

Crystal structure: contains datablock(s) I, global. DOI: 10.1107/S1600536812042936/go2065sup1.cif

Structure factors: contains datablock(s) I. DOI: 10.1107/S1600536812042936/go2065Isup2.hkl

Supplementary material file. DOI: 10.1107/S1600536812042936/go2065Isup3.cml

Additional supplementary materials:  crystallographic information; 3D view; checkCIF report

## Figures and Tables

**Table 1:** Hydrogen-bond geometry (Å, °)

*D*—H⋯*A*	*D*—H	H⋯*A*	*D*⋯*A*	*D*—H⋯*A*
N3—H3*A*⋯O1^i^	0.85 (3)	2.16 (3)	2.982 (3)	161 (2)
C18—H18⋯O1^i^	0.93	2.58	3.335 (3)	138
C23—H23*C*⋯O1^ii^	0.96	2.59	3.512 (3)	162

Symmetry codes: (i) 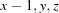; (ii) 
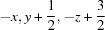
.

**Fig. 1.**
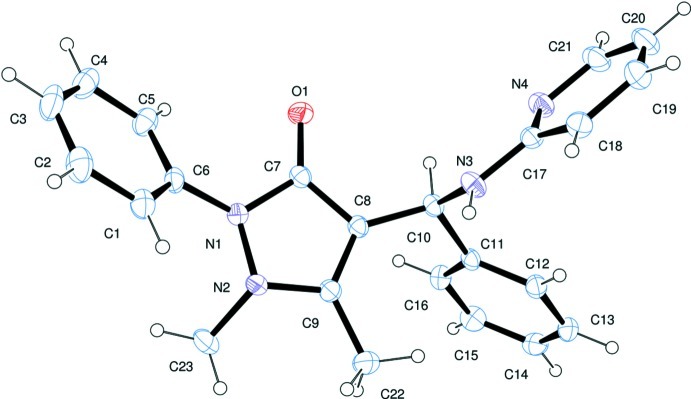
The structure of APBA, showing 30% probability displacement ellipsoids.

**Fig. 2.**
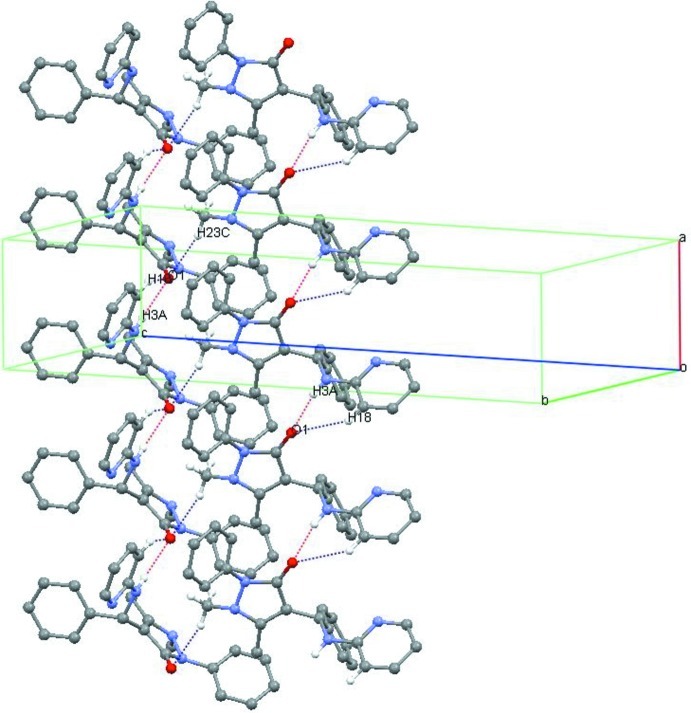
Part of the crystal structure of APBA, showing the hydrogen bonding patterns. For the sake of clarity, only H atoms involved in hydrogen bonding are shown. Dashed lines represent hydrogen bonds.

## supplementary crystallographic information

### Comment

Antipyrine (2,3-dimethyl-1-phenylpyrazol-5-one) is one of the best known pyrazole derivatives used as pain-relieving medicine. It is the first pyrazole derivative to be introduced as an analgesic and antipyretic drug. Antipyrine and its derivatives have been long known for their wide spectrum of biological activities. Their roles in biological processes have become a topic of study in recent years. Though rarely used alone, nowadays, on account of its toxicity, antipyrine forms part of some combination products used as pain-relieving medicines. Many of its derivatives like amidopyrine and metamizol are well known and widely used analgesics. Therefore a detailed knowledge of the structure and the possible modes of interaction of antipyrine are important in elucidating the molecular mechanism of the action of pain-relieving medicines. The crystal structure of antipyrine, its derivatives and some of its metallic complexes have already been reported (see related literature). Presently, the crystal structure of 2-amino pyridino benzyl antipyrine {1,2-dihydro-2,3-dimethyl-1-phenyl-4-(phenyl (pyridin-2-yl amino) methyl)pyrazol-5-one} has been elucidated. The conformational features and the hydrogen bonding analysis have been analysed in order to have a further insight in to their chemical and pharmaceutical aspects. The chemical scheme of 2-amino pyridino benzyl antipyrine is shown.

An *ORTEP-3* (Farrugia, 1997) diagram of the asymmetric unit of 2-amino pyridino benzyl antipyrine (APBA) is shown in Figure 1. In APBA, the antipyrine, benzene and pyridyl amine molecules are bridged by C10 which is the asymmetric sp3 carbon in the structure. Bond lengths and angles are comparable with those of the related structures reported. Four flat fragments are present in the structure elucidated. The dihedral angles between these flat rings A (C1—C6), B (N1, N2, C7—C9), C (C11—C16) and D (C17—C21, N4) are A/B= 45.57 (11)°, A/C= 54.96 (10)°, A/D= 82.10 (11)°, B/C= 83.98 (10)°, B/D= 67.85 (10)° and C/D= 80.15 (10)°. These values show that the pyrazole (B), benzene (C) and pyridyl (D) rings are almost perpendicular to each other. The crystal packing of the compound APBA is stabilized by N3–H3A···O1 and C18—H18···O1 hydrogen bonds which link the molecules into one-dimensional chains which run parallel to the a-axis, Table 1, Figure 2. In addition there is a short contact C23–H23C···O1.

### Experimental

5.31 mmol of 2-aminopyridine and 5.31 mmol of benzaldehyde were dissolved in ethanol solution. To this mixture 5.31 mmol of antipyrine was added and stirred well. The contents were refluxed at a temperature of 333 K for 12 h. The product obtained by Mannich base condensation was filtered, dried and then washed with distilled water. It is then dried in the air oven at 323 K. By slow evaporation technique, using ethanol, orange colour block shaped crystals of title compound suitable for X-ray diffraction analysis were obtained from the product.

### Refinement

For APBA, which crystallizes in the space group *P*2_1_2_1_2_1_, the Friedel equivalents were merged in the absence of significant anomalous scattering effects prior to the final refinement cycles and the absolute structure was assigned arbitrarily. The 002 reflection was omitted because it was obscured by the beamstop. The H atom attached to N3 was refined isotropically. H atoms attached to C atoms were included in calculated positions and treated as riding atoms, with C—H=0.93 to 0.96Å, and with *U*_iso_(H)=1.5Ueq(C) for those attached to methyl H atoms and *U*_iso_(H)=1.2Ueq(C) for the remaining H atoms. The positions of the methyl hydrogens and that attached to N3 were checked on a final difference map.

### Crystal data

**Table:**

C_23_H_22_N_4_O	*F*(000) = 784
*M**_r_* = 370.45	*D*_x_ = 1.293 Mg m^−^^3^
Orthorhombic, *P*2_1_2_1_2_1_	Mo *K*α radiation, λ = 0.71073 Å
Hall symbol: P 2ac 2ab	Cell parameters from 3758 reflections
*a* = 5.701 (5) Å	θ = 2.2–25.4°
*b* = 12.485 (5) Å	µ = 0.08 mm^−^^1^
*c* = 26.736 (5) Å	*T* = 273 K
*V* = 1903.0 (19) Å^3^	Block, orange
*Z* = 4	0.3 × 0.2 × 0.2 mm

### Data collection

**Table:**

Bruker Kappa APEXII CCD diffractometer	2337 reflections with *I* > 2σ(*I*)
Graphite monochromator	*R*_int_ = 0.036
ω and φ scan	θ_max_ = 29.5°, θ_min_ = 2.2°
Absorption correction: multi-scan (*SADABS*; Bruker, 1999)	*h* = −4→7
*T*_min_ = 0.976, *T*_max_ = 0.984	*k* = −17→17
14432 measured reflections	*l* = −36→34
2959 independent reflections	

### Refinement

**Table:**

Refinement on *F*^2^	Primary atom site location: structure-invariant direct methods
Least-squares matrix: full	Secondary atom site location: difference Fourier map
*R*[*F*^2^ > 2σ(*F*^2^)] = 0.040	Hydrogen site location: inferred from neighbouring sites
*wR*(*F*^2^) = 0.095	H atoms treated by a mixture of independent and constrained refinement
*S* = 1.01	*w* = 1/[σ^2^(*F*_o_^2^) + (0.0435*P*)^2^ + 0.2362*P*] where *P* = (*F*_o_^2^ + 2*F*_c_^2^)/3
2959 reflections	(Δ/σ)_max_ < 0.001
259 parameters	Δρ_max_ = 0.18 e Å^−^^3^
0 restraints	Δρ_min_ = −0.19 e Å^−^^3^

### Special details

**Table:**

Geometry. All e.s.d.'s (except the e.s.d. in the dihedral angle between two l.s. planes) are estimated using the full covariance matrix. The cell e.s.d.'s are taken into account individually in the estimation of e.s.d.'s in distances, angles and torsion angles; correlations between e.s.d.'s in cell parameters are only used when they are defined by crystal symmetry. An approximate (isotropic) treatment of cell e.s.d.'s is used for estimating e.s.d.'s involving l.s. planes.
Refinement. Refinement of *F*^2^ against ALL reflections. The weighted *R*-factor *wR* and goodness of fit *S* are based on *F*^2^, conventional *R*-factors *R* are based on *F*, with *F* set to zero for negative *F*^2^. The threshold expression of *F*^2^ > σ(*F*^2^) is used only for calculating *R*-factors(gt) *etc*. and is not relevant to the choice of reflections for refinement. *R*-factors based on *F*^2^ are statistically about twice as large as those based on *F*, and *R*- factors based on ALL data will be even larger.

### Fractional atomic coordinates and isotropic or equivalent isotropic displacement parameters (Å^2^)

**Table:**

	*x*	*y*	*z*	*U*_iso_*/*U*_eq_	
O1	0.3555 (2)	0.08528 (11)	0.70108 (5)	0.0391 (3)	
N1	0.2298 (3)	0.21846 (12)	0.75511 (6)	0.0315 (4)	
N2	0.0380 (3)	0.28913 (12)	0.75618 (6)	0.0335 (4)	
N3	−0.2190 (3)	0.08348 (13)	0.63621 (6)	0.0352 (4)	
N4	−0.0868 (3)	−0.01040 (13)	0.56735 (6)	0.0396 (4)	
C1	0.1607 (4)	0.17086 (18)	0.84215 (8)	0.0405 (5)	
H1	0.0148	0.2041	0.8409	0.049*	
C2	0.2324 (5)	0.11982 (18)	0.88527 (9)	0.0508 (6)	
H2	0.1348	0.1191	0.9131	0.061*	
C3	0.4462 (5)	0.0702 (2)	0.88728 (9)	0.0551 (7)	
H3	0.492	0.0342	0.9161	0.066*	
C4	0.5923 (5)	0.07368 (19)	0.84676 (9)	0.0505 (6)	
H4	0.7383	0.0407	0.8484	0.061*	
C5	0.5254 (4)	0.12577 (16)	0.80329 (9)	0.0409 (5)	
H5	0.6266	0.1293	0.7761	0.049*	
C6	0.3058 (4)	0.17246 (15)	0.80100 (7)	0.0325 (4)	
C7	0.2224 (3)	0.16029 (14)	0.71041 (7)	0.0290 (4)	
C8	0.0321 (3)	0.20467 (14)	0.68212 (7)	0.0280 (4)	
C9	−0.0668 (4)	0.28329 (14)	0.71018 (7)	0.0313 (4)	
C10	−0.0332 (3)	0.16209 (15)	0.63147 (7)	0.0298 (4)	
H10	0.1045	0.1232	0.6192	0.036*	
C11	−0.0829 (3)	0.25022 (15)	0.59317 (7)	0.0282 (4)	
C12	−0.2876 (4)	0.25263 (16)	0.56545 (7)	0.0331 (4)	
H12	−0.4044	0.2023	0.5715	0.04*	
C13	−0.3200 (4)	0.32958 (17)	0.52860 (8)	0.0390 (5)	
H13	−0.458	0.3303	0.51	0.047*	
C14	−0.1491 (4)	0.40456 (17)	0.51947 (8)	0.0410 (5)	
H14	−0.1703	0.4559	0.4947	0.049*	
C15	0.0535 (4)	0.40313 (17)	0.54730 (8)	0.0420 (5)	
H15	0.1691	0.4541	0.5414	0.05*	
C16	0.0869 (4)	0.32708 (16)	0.58385 (7)	0.0355 (4)	
H16	0.2247	0.3273	0.6025	0.043*	
C17	−0.2525 (4)	0.00363 (14)	0.60187 (7)	0.0319 (4)	
C18	−0.4543 (4)	−0.06045 (16)	0.60472 (8)	0.0400 (5)	
H18	−0.5674	−0.0482	0.6291	0.048*	
C19	−0.4802 (5)	−0.14120 (17)	0.57079 (9)	0.0497 (6)	
H19	−0.6112	−0.1855	0.572	0.06*	
C20	−0.3093 (5)	−0.15665 (18)	0.53444 (9)	0.0527 (7)	
H20	−0.3239	−0.2107	0.5107	0.063*	
C21	−0.1212 (5)	−0.09074 (17)	0.53461 (8)	0.0473 (6)	
H21	−0.007	−0.1017	0.5103	0.057*	
C22	−0.2613 (4)	0.35812 (17)	0.69792 (9)	0.0460 (6)	
H22A	−0.1975	0.4263	0.6884	0.069*	
H22B	−0.36	0.367	0.7267	0.069*	
H22C	−0.3518	0.3294	0.6708	0.069*	
C23	0.0838 (5)	0.39199 (16)	0.78084 (9)	0.0508 (6)	
H23A	0.1981	0.4314	0.762	0.076*	
H23B	0.1424	0.3794	0.814	0.076*	
H23C	−0.0591	0.4325	0.7827	0.076*	
H3A	−0.332 (5)	0.0995 (19)	0.6557 (9)	0.049 (7)*	

### Atomic displacement parameters (Å^2^)

**Table:**

	*U*^11^	*U*^22^	*U*^33^	*U*^12^	*U*^13^	*U*^23^
O1	0.0394 (8)	0.0403 (8)	0.0375 (8)	0.0110 (7)	0.0007 (7)	−0.0041 (6)
N1	0.0361 (9)	0.0307 (8)	0.0278 (9)	0.0034 (7)	−0.0035 (7)	−0.0019 (6)
N2	0.0436 (9)	0.0287 (8)	0.0283 (9)	0.0074 (7)	−0.0037 (7)	−0.0045 (6)
N3	0.0436 (10)	0.0328 (8)	0.0292 (9)	−0.0055 (8)	0.0075 (8)	−0.0056 (7)
N4	0.0508 (11)	0.0353 (9)	0.0327 (9)	0.0003 (9)	0.0028 (9)	−0.0044 (7)
C1	0.0454 (12)	0.0427 (11)	0.0335 (12)	−0.0056 (10)	−0.0035 (10)	0.0010 (9)
C2	0.0648 (16)	0.0552 (14)	0.0323 (12)	−0.0131 (14)	−0.0021 (12)	0.0063 (10)
C3	0.0689 (17)	0.0550 (14)	0.0414 (14)	−0.0137 (14)	−0.0234 (13)	0.0140 (11)
C4	0.0433 (13)	0.0489 (14)	0.0594 (16)	−0.0064 (11)	−0.0209 (12)	0.0106 (11)
C5	0.0359 (11)	0.0433 (12)	0.0434 (13)	−0.0057 (9)	−0.0056 (10)	0.0067 (9)
C6	0.0388 (11)	0.0305 (9)	0.0283 (10)	−0.0070 (8)	−0.0088 (9)	0.0000 (7)
C7	0.0328 (10)	0.0284 (9)	0.0257 (10)	−0.0024 (8)	0.0024 (8)	−0.0005 (7)
C8	0.0337 (10)	0.0271 (9)	0.0232 (9)	−0.0015 (8)	0.0000 (7)	0.0007 (7)
C9	0.0390 (10)	0.0279 (9)	0.0269 (10)	0.0009 (8)	−0.0015 (8)	0.0018 (7)
C10	0.0334 (9)	0.0310 (9)	0.0248 (9)	−0.0008 (8)	0.0008 (8)	−0.0026 (7)
C11	0.0338 (9)	0.0309 (9)	0.0200 (9)	0.0000 (8)	0.0012 (8)	−0.0035 (7)
C12	0.0328 (10)	0.0361 (9)	0.0305 (11)	−0.0005 (9)	−0.0017 (8)	−0.0032 (8)
C13	0.0420 (12)	0.0446 (11)	0.0304 (11)	0.0070 (10)	−0.0078 (9)	−0.0045 (9)
C14	0.0567 (14)	0.0375 (11)	0.0288 (11)	0.0090 (11)	0.0039 (10)	0.0052 (8)
C15	0.0476 (13)	0.0390 (11)	0.0396 (12)	−0.0055 (11)	0.0071 (10)	0.0057 (9)
C16	0.0335 (10)	0.0400 (11)	0.0330 (11)	−0.0036 (9)	−0.0023 (8)	0.0006 (8)
C17	0.0415 (10)	0.0263 (9)	0.0278 (10)	0.0024 (8)	−0.0037 (8)	0.0026 (7)
C18	0.0429 (12)	0.0348 (10)	0.0423 (13)	−0.0010 (9)	−0.0038 (10)	0.0007 (8)
C19	0.0548 (14)	0.0384 (12)	0.0558 (15)	−0.0077 (11)	−0.0115 (13)	−0.0040 (10)
C20	0.0776 (18)	0.0353 (11)	0.0452 (14)	0.0009 (13)	−0.0092 (13)	−0.0140 (10)
C21	0.0679 (16)	0.0385 (11)	0.0354 (12)	0.0068 (12)	0.0022 (11)	−0.0090 (9)
C22	0.0517 (13)	0.0461 (12)	0.0403 (13)	0.0179 (11)	−0.0038 (11)	−0.0047 (9)
C23	0.0748 (17)	0.0324 (11)	0.0452 (13)	0.0050 (12)	−0.0110 (13)	−0.0129 (9)

### Geometric parameters (Å, º)

**Table:**

O1—C7	1.231 (2)	C10—H10	0.98
N1—C7	1.399 (2)	C11—C12	1.383 (3)
N1—N2	1.405 (2)	C11—C16	1.386 (3)
N1—C6	1.422 (2)	C12—C13	1.389 (3)
N2—C9	1.369 (2)	C12—H12	0.93
N2—C23	1.467 (2)	C13—C14	1.373 (3)
N3—C17	1.369 (2)	C13—H13	0.93
N3—C10	1.450 (3)	C14—C15	1.374 (3)
N3—H3A	0.85 (3)	C14—H14	0.93
N4—C17	1.332 (3)	C15—C16	1.376 (3)
N4—C21	1.346 (3)	C15—H15	0.93
C1—C6	1.377 (3)	C16—H16	0.93
C1—C2	1.379 (3)	C17—C18	1.403 (3)
C1—H1	0.93	C18—C19	1.364 (3)
C2—C3	1.368 (4)	C18—H18	0.93
C2—H2	0.93	C19—C20	1.390 (4)
C3—C4	1.367 (4)	C19—H19	0.93
C3—H3	0.93	C20—C21	1.352 (4)
C4—C5	1.385 (3)	C20—H20	0.93
C4—H4	0.93	C21—H21	0.93
C5—C6	1.382 (3)	C22—H22A	0.96
C5—H5	0.93	C22—H22B	0.96
C7—C8	1.434 (3)	C22—H22C	0.96
C8—C9	1.358 (3)	C23—H23A	0.96
C8—C10	1.502 (3)	C23—H23B	0.96
C9—C22	1.486 (3)	C23—H23C	0.96
C10—C11	1.530 (3)		
			
C7—N1—N2	108.65 (15)	C12—C11—C10	122.07 (17)
C7—N1—C6	122.45 (15)	C16—C11—C10	119.27 (17)
N2—N1—C6	118.22 (16)	C11—C12—C13	120.52 (19)
C9—N2—N1	106.73 (15)	C11—C12—H12	119.7
C9—N2—C23	121.87 (16)	C13—C12—H12	119.7
N1—N2—C23	114.84 (18)	C14—C13—C12	120.2 (2)
C17—N3—C10	122.43 (17)	C14—C13—H13	119.9
C17—N3—H3A	118.4 (17)	C12—C13—H13	119.9
C10—N3—H3A	116.5 (17)	C13—C14—C15	119.4 (2)
C17—N4—C21	116.5 (2)	C13—C14—H14	120.3
C6—C1—C2	119.8 (2)	C15—C14—H14	120.3
C6—C1—H1	120.1	C14—C15—C16	120.7 (2)
C2—C1—H1	120.1	C14—C15—H15	119.7
C3—C2—C1	120.4 (2)	C16—C15—H15	119.7
C3—C2—H2	119.8	C15—C16—C11	120.6 (2)
C1—C2—H2	119.8	C15—C16—H16	119.7
C4—C3—C2	119.8 (2)	C11—C16—H16	119.7
C4—C3—H3	120.1	N4—C17—N3	117.50 (19)
C2—C3—H3	120.1	N4—C17—C18	122.95 (18)
C3—C4—C5	120.8 (2)	N3—C17—C18	119.54 (19)
C3—C4—H4	119.6	C19—C18—C17	118.3 (2)
C5—C4—H4	119.6	C19—C18—H18	120.9
C6—C5—C4	119.0 (2)	C17—C18—H18	120.9
C6—C5—H5	120.5	C18—C19—C20	119.4 (2)
C4—C5—H5	120.5	C18—C19—H19	120.3
C1—C6—C5	120.20 (19)	C20—C19—H19	120.3
C1—C6—N1	120.80 (19)	C21—C20—C19	118.0 (2)
C5—C6—N1	118.96 (19)	C21—C20—H20	121
O1—C7—N1	123.31 (17)	C19—C20—H20	121
O1—C7—C8	130.83 (18)	N4—C21—C20	124.9 (2)
N1—C7—C8	105.83 (16)	N4—C21—H21	117.6
C9—C8—C7	107.59 (17)	C20—C21—H21	117.6
C9—C8—C10	130.63 (19)	C9—C22—H22A	109.5
C7—C8—C10	121.76 (17)	C9—C22—H22B	109.5
C8—C9—N2	110.70 (17)	H22A—C22—H22B	109.5
C8—C9—C22	129.95 (19)	C9—C22—H22C	109.5
N2—C9—C22	119.35 (17)	H22A—C22—H22C	109.5
N3—C10—C8	109.99 (15)	H22B—C22—H22C	109.5
N3—C10—C11	114.21 (16)	N2—C23—H23A	109.5
C8—C10—C11	113.27 (15)	N2—C23—H23B	109.5
N3—C10—H10	106.2	H23A—C23—H23B	109.5
C8—C10—H10	106.2	N2—C23—H23C	109.5
C11—C10—H10	106.2	H23A—C23—H23C	109.5
C12—C11—C16	118.57 (18)	H23B—C23—H23C	109.5
			
C7—N1—N2—C9	−7.28 (19)	N1—N2—C9—C22	−173.48 (17)
C6—N1—N2—C9	−152.75 (17)	C23—N2—C9—C22	−38.8 (3)
C7—N1—N2—C23	−145.59 (17)	C17—N3—C10—C8	−154.18 (18)
C6—N1—N2—C23	68.9 (2)	C17—N3—C10—C11	77.2 (2)
C6—C1—C2—C3	−0.4 (3)	C9—C8—C10—N3	−83.2 (2)
C1—C2—C3—C4	1.7 (4)	C7—C8—C10—N3	94.7 (2)
C2—C3—C4—C5	−0.8 (4)	C9—C8—C10—C11	46.0 (3)
C3—C4—C5—C6	−1.4 (3)	C7—C8—C10—C11	−136.16 (18)
C2—C1—C6—C5	−1.8 (3)	N3—C10—C11—C12	−0.6 (2)
C2—C1—C6—N1	175.87 (19)	C8—C10—C11—C12	−127.61 (19)
C4—C5—C6—C1	2.7 (3)	N3—C10—C11—C16	−177.14 (17)
C4—C5—C6—N1	−175.04 (19)	C8—C10—C11—C16	55.9 (2)
C7—N1—C6—C1	−121.8 (2)	C16—C11—C12—C13	0.9 (3)
N2—N1—C6—C1	18.7 (3)	C10—C11—C12—C13	−175.58 (18)
C7—N1—C6—C5	55.9 (3)	C11—C12—C13—C14	−0.3 (3)
N2—N1—C6—C5	−163.60 (16)	C12—C13—C14—C15	−0.4 (3)
N2—N1—C7—O1	−173.02 (17)	C13—C14—C15—C16	0.4 (3)
C6—N1—C7—O1	−29.3 (3)	C14—C15—C16—C11	0.2 (3)
N2—N1—C7—C8	5.19 (19)	C12—C11—C16—C15	−0.9 (3)
C6—N1—C7—C8	148.89 (17)	C10—C11—C16—C15	175.72 (18)
O1—C7—C8—C9	176.9 (2)	C21—N4—C17—N3	178.70 (18)
N1—C7—C8—C9	−1.2 (2)	C21—N4—C17—C18	−0.2 (3)
O1—C7—C8—C10	−1.4 (3)	C10—N3—C17—N4	10.0 (3)
N1—C7—C8—C10	−179.44 (16)	C10—N3—C17—C18	−171.04 (18)
C7—C8—C9—N2	−3.4 (2)	N4—C17—C18—C19	0.6 (3)
C10—C8—C9—N2	174.63 (18)	N3—C17—C18—C19	−178.4 (2)
C7—C8—C9—C22	176.7 (2)	C17—C18—C19—C20	−0.8 (3)
C10—C8—C9—C22	−5.2 (4)	C18—C19—C20—C21	0.7 (4)
N1—N2—C9—C8	6.6 (2)	C17—N4—C21—C20	0.1 (3)
C23—N2—C9—C8	141.4 (2)	C19—C20—C21—N4	−0.3 (4)

### Hydrogen-bond geometry (Å, º)

**Table:**

*D*—H···*A*	*D*—H	H···*A*	*D*···*A*	*D*—H···*A*
N3—H3*A*···O1^i^	0.85 (3)	2.16 (3)	2.982 (3)	161 (2)
C18—H18···O1^i^	0.93	2.58	3.335 (3)	138
C23—H23*C*···O1^ii^	0.96	2.59	3.512 (3)	162

Symmetry codes: (i) *x*−1, *y*, *z*; (ii) −*x*, *y*+1/2, −*z*+3/2.
